# Squamous cell carcinoma of the external auditory canal: A case report and review of the literature

**DOI:** 10.3892/ol.2013.1241

**Published:** 2013-03-08

**Authors:** KOPPANY VISNYEI, RUPINDER GILL, EFAT AZIZI, BRUCE CULLINEY

**Affiliations:** 1 Departments of Internal Medicine, Beth Israel Medical Center, Albert Einstein College of Medicine, New York, NY 1003, USA; 2 Pain Medicine and Palliative Care, Beth Israel Medical Center, Albert Einstein College of Medicine, New York, NY 1003, USA; 3 Division of Hematology Oncology, Beth Israel Medical Center, Albert Einstein College of Medicine, New York, NY 1003, USA

**Keywords:** squamous cell carcinoma, external auditory canal, middle ear, Pittsburgh classification, quad shot

## Abstract

Squamous cell carcinoma of the external auditory canal, middle ear and temporal bone is a rare and unusual malignancy. The lack of a unifying classification system in the past, along with the rarity of the disease has made the development of clear treatment guidelines difficult. In this report, we describe a clinical case of a patient with this rare malignancy, discuss the challenges associated with the diagnosis and treatment of the disease, and review the literature for trends while outlining the most beneficial treatment strategy for this patient population.

## Introduction

The ear canal, middle ear and temporal bone are rare sites of malignancies among which squamous cell carcinoma is the most commonly occurring cancer type (1). The incidence of this tumor in the US is estimated to be only 0.1–0.6/100,000 population/year. However, the challenging anatomical location and invasive nature makes this type of cancer extremely difficult to treat, particularly in more advanced stages (2). Although several treatment modalities have been described in the literature, there is a lack of consensus as to the best treatment, mainly due to the absence of prospective randomized studies (3). The most frequently reported treatment involves surgical resection with or without adjuvant radiotherapy. Chemotherapy, brachytherapy or alternative treatment methods such as superselective intra-arterial chemotherapy injection have been described in the literature, however, their exact role remains to be determined (4,5).

While there is a lack of data for optimal tumor treatment, a convincing body of evidence has shown that early stage cancer is associated with a higher treatment success and survival rate, compared to late stage disease (3,6). Therefore, the most important factors in treating patients with this type of malignancy are early detection and diagnosis. However, since many patients present with non-specific and unclear signs of chronic inflammation and infection, detection and diagnosis of this malignant type is difficult. Additionally, chronic and recurring infections, thought to often precede tumor development, can lead to decreased follow-up motivation, resulting in a delay in diagnosis.

In light of these diagnostic and therapeutic challenges, the present report described a case of advanced squamous cell carcinoma of the external auditory canal in a patient whose cancer was initially diagnosed and treated as osteomyelitis, in the setting of chronic ear infections, at a non-US institution.

## Case report

A 73-year-old Hispanic female with a past medical history of diabetes and chronic left-sided suppurative otitis media that resulted in mastoidectomy in her mid-thirties, was admitted to our institution with left-sided otalgia. The pain was associated with a serosanguineous ear discharge, dizziness, headache, fever, sore throat, generalized weakness and a twenty-pound unintentional weight loss. Previously, the patient had been diagnosed and treated for chronic mastoiditis and later for temporomandibular joint osteomyelitits that extended to the temporal bone. She received several courses of antibiotics, without relief. At the time, cultures of the ear grew staphylococcus epidermidis and diphteroid species. Left ear canal biopsy revealed a small number of keratinizing atypical squamous cells and chronic inflammation, suspicious for neoplasia.

**Figure 1:**
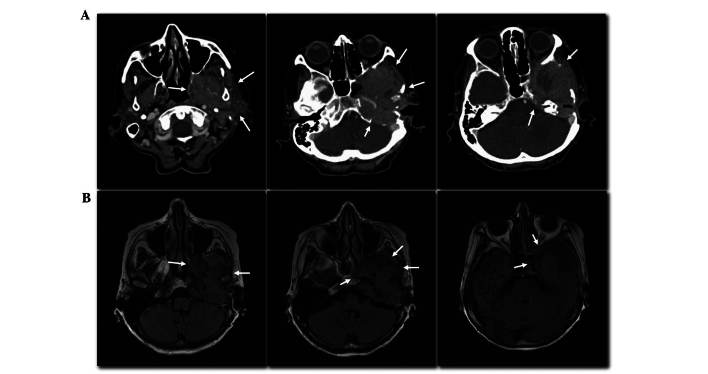
(A) Contrast-enhanced computed tomography (CT) imaging of the head shows a large soft tissue mass extending to the floor of the left middle cranial fossa with mild mass effect on the inferior temporal lobe. Destruction of the left mandibular condyle, left anterior temporal calvarium and the petrous of the left temporal bone are evident. The mass has eliminated the left jugular bulb and extends to the left cavernous sinus and the lateral wall of the orbit. (B) Magnetic resonance imaging (MRI) imaging verifies the involvement of the left cavernous sinus and the dura along the sphenoid and temporal bones. The left carotid artery shows significant narrowing suggesting circumferential involvement. The left pterygoid muscle and temporomandibular joint are also involved in the malignant process. The images suggest intraorbital extension of the tumor.

On admission to our institution, physical examination revealed serosanguineous discharge from the left external ear canal as well as tenderness of the left mastoid process, the temporomandibular joint and the submandibular region. The patient’s symptoms were associated with left-sided diffuse facial swelling and signs consistent with ipsilateral facial nerve palsy. Basic laboratory work-up revealed a slightly elevated white blood cell count of 12.2×10^6^/*μ*l, but otherwise normal laboratory parameters. Cultures of the blood and ear discharge were both negative. The chest roentgenogram was within normal limits. Computed tomography (CT) of the head with and without contrast, revealed a soft tissue mass invading the left middle cranial fossa with destruction of the adjacent sphenoid and temporal bones (Fig. 1A). Magnetic resonance imaging (MRI) of the brain with and without contrast, revealed an enhancing, expansive and erosive lesion in the same area with invasion of the left cavernous sinus (Fig. 1B). Fine needle aspiration of the mass in left middle cranial fossa identified well-differentiated squamous cell carcinoma. CT of the chest, abdomen and pelvis were negative for metastatic disease.

Based on the Pittsburgh staging system, the diagnosis of a stage IV squamous cell carcinoma was made (7). Considering the advanced stage, the patient was deemed not to be a surgical candidate. She received a course of specific palliative radio-therapy for advanced head and neck cancer (Quad shot) (8), with concurrent carboplatin radiosensitization. This treatment consisted of intensity-modulated radiation treatment administered twice daily, 2 days per week. Over an 8-week period, the left external auditory canal was exposed to a total dose of 29.6 Gy, given in 8 fractions, followed by radiation to the left middle ear with a total of 14.8 Gy, given in 4 fractions. Minimal improvement was noted in the patient’s symptoms over a short period of time, prior to return of a steady decline that eventually led to the patient succumbing to the disease.

## Discussion

Squamous cell carcinoma of the external auditory meatus, middle ear and temporal bone is an unusual and rare malignancy, which may explain the fact that there is no American Joint Committee on Cancer (AJCC) or Union for International Cancer Control (UICC) staging system for this type of neoplasm. Arriaga *et al*(7) suggested a staging system in 1990, which has since entered the literature as the Pittsburgh staging system, allowing for a more accurate comparison of treatment and outcomes in patients with this disease. This system underwent minor revision by Moody *et al*(9) in 2000 (Table I).

Although the lack of a unifying classification, along with the rarity of the disease have made the development of clear treatment guidelines difficult, there have been uniform observations in the literature, that may help us outline the most beneficial treatment strategies for this patient population.

Surgical resection is crucial as a treatment modality, and early surgical intervention is associated with increased survival (9–11). Additionally, different stages of the disease may require a different level of surgical resection.

Tumors that are limited to the external auditory canal with or without limited bone erosion and soft tissue involvement are classified as Pittsburgh stages T1 and T2 (Table I). In these stages, local resection of the external auditory canal does not seem to be sufficient (12). Mastoidectomy, lateral temporal bone resection (TBR) and subtotal TBR are more appropriate and these techniques showed similar survival in a retrospective review including 144 patients, with a five-year survival of 50, 48.6 and 50%, respectively (13). The same study did not find evidence of improved survival with the addition of radiation therapy (RT) to TBR (with 48 vs. 44.4% 5-year survival for TBR + RT vs. TBR alone, respectively) (13). In a retrospective analysis of 21 patients, Kollert *et al*(12) found that stage-dependent lateral or subtotal TBR combined with parotidectomy as well as a neck dissection was the most beneficial approach. However, the role of chemotherapy remains to be determined. Ogawa *et al*(14) suggest that it does not affect disease-free survival (DFS). Authors of that study found the 5-year DFS rate in T1, T2 and T3 patients to be 83, 45 and 0% in the RT group (P<0.0001) and 75, 75 and 46% in the group that underwent surgery with RT (P=0.13). Based on those results, they recommend radical radiotherapy alone as the treatment of choice for early-stage (T1) disease and surgery with radiotherapy for more advanced (T2-3) disease (14).

In Pittsburgh stage T3 disease, i.e., with middle ear extension, Kollert *et al*(12) again recommend stage-dependent lateral or subtotal TBR combined with parotidectomy and neck dissection (12). Prasad *et al*(13) noted that subtotal TBR appears to be more beneficial than lateral TBR or mastoidectomy, with 42 vs. 29 vs. 17% 5-year survival. The same study showed clear survival benefits from additional RT only in patients who underwent mastoidectomy (20 vs. 0% 5-year survival). However, the benefits of RT could not be estimated in patients with lateral or total TBR due to lack of data (13). While some investigators suggest RT for recurrent cases, questionable free margins and/or lymph node metastases (12), other authors recommend surgery with RT as the standard of care in general for all advanced disease (T2-3) (14).

Involvement of the petrous apex, cochlea, carotid, jugular foramen, dura, temporomandibular joint or the styloid process as well as the presence of facial paresis signal Pittsburgh stage T4 disease (Table I). In case of dura mater involvement, resection of the affected dura does not appear to improve survival. Furthermore, whether surgical resection of the involved petrous apex, brain parenchyma or internal carotid artery is of any survival benefit remains unclear (13). However, surgery remains important in advanced disease. In cases where T4 lesions did not involve the pyramidal apex, carotid canal, dura or any lymph nodes, surgical intervention was found to improve the estimated survival rate to a level as good as that of T3 lesions (15). Similarly, in a retrospective case review of 12 patients, the 5-year estimated survival improved up to 75% for T4 tumors after surgery versus 16% for patients who did not undergo surgery, (15). In T4 disease, carotid and middle- or posterior fossa invasion are considered to be unresectable (1).

Irrespective of stage of the disease, Moffat *et al*(11) emphasized the importance of early referral and aggressive primary surgical treatment with post-operative radiotherapy, in a retrospective analysis of 39 patients. Another important factor is complete resection with clear surgical margins. Several retrospective analyses showed increased survival rates with tumor-free resection margins, in all stages of disease (12,14–17). However, our patient did not qualify as a surgical candidate due to her advanced stage at presentation.

There is conflicting data in the literature regarding the benefits of chemotherapy with or without radiation. While Ogawa *et al*(14) did not find chemotherapy to increase DFS in any stage of the disease, a multi-institutional review by Yin *et al*(17) has suggested increased survival with chemotherapy in stage 3 and 4 disease, if combined with surgery and radiation (28.7 vs. 52.5%; surgery + radiation vs. surgery + radiation + chemotherapy).

In a small study (18) that evaluated six patients who underwent radiation with total doses ranging from 30 to 88 Gy, two patients who received doses over 60 Gy survived for >5 years, compared to a mean survival of 3 years and 11 months. The authors of that study concluded that tumor doses of at least 70 Gy may be necessary for local control of advanced disease (18). While Quad shot therapy was validated in a phase II trial as a palliative approach for advanced head and neck cancers, it has not been specifically studied in external auditory canal carcinomas (8). However, this approach has been shown to have a response rate in >50% of patients with advanced head and neck cancers. This treatment can be delivered with minimal toxicity and has been shown to significantly improve the quality of life (8). While our patient had minimal benefits from Quad shot therapy, larger scale studies are necessary to define the exact role of this and alternative radiation regiments with or without chemotherapy.

Predictors of poor survival in this patient population include extensive tumor involvement, neck node metastasis, facial nerve paralysis, pain, middle ear involvement, cervical or periparotid lymphadenopathy and concomitant chronic otitis media (1,7,15,19). Advanced stage disease, node-positive disease, positive surgical margins, tumor recurrence, poorly differentiated squamous cell histological findings, brain involvement and salvage surgery were also associated with a poorer outcome (11,17). Possible predisposing factors for the disease are preceding head and neck radiation for nasopharyngeal and skin neoplasms (1).

In conclusion, in this report, we describe a case of a rare and aggressive tumor type for which the most beneficial therapeutic approach remains to be determined. It is clear however, that, if diagnosed late, this disease exhibits poor outcomes, while it is associated with higher response rates and increased survival at early stages.

Thus, emphasis should be placed on the importance of early detection, diagnosis and treatment of squamous cell carcinoma of the temporal bone and middle ear as the simplest and most effective measure to increase patient survival. We also urge the medical community for prompt diagnostic work-up in patients with chronic and treatment-resistant ear infections.

## Figures and Tables

**Table I:** The Pittsburgh classification system for external auditory meatus carcinoma.

Stage	Status
T1	Tumor limited to EAM without bony erosion or evidence of soft tissue extension
T2	Limited EAM erosion (not full thickness), or radiographic findings consistent with limited (<5 mm) soft tissue involvement
T3	Erosion into the EAM (full thickness) with limited (<5 mm) soft tissue involvement, or tumor involving the middle ear and/or mastoid, or presence of facial paralysis
T4	Tumor eroding the cochlea, petrous apex, medial wall of middle ear, carotid canal, jugular foramen or dura, or with extensive (>5 mm) soft tissue involvement
N	As described by the American Joint Committee for classifying lymph node involvement in head and neck neoplasms. However, any node involvement is considered to be advanced disease: stage III, T1, N1; stage IV, T2, T3, T4, N1
M	Any metastasis is considered to be advanced disease: stage IV, M1

EAM, external auditory meatus.
